# Effects of Substitution on Solid-State Fluorescence in 9-Aryl-9-methyl-9*H*-9-silafluorenes

**DOI:** 10.3390/molecules21091173

**Published:** 2016-09-03

**Authors:** Yoshinori Yamanoi, Takayuki Nakashima, Masaki Shimada, Hiroaki Maeda, Hiroshi Nishihara

**Affiliations:** Department of Chemistry, School of Science, The University of Tokyo, 7-3-1 Hongo, Bunkyo-ku, Tokyo 113-0033, Japan; k-nakashima@chem.s.u-tokyo.ac.jp (T.N.); shimada@chem.s.u-tokyo.ac.jp (M.S.); h-maeda@chem.s.u-tokyo.ac.jp (H.M.)

**Keywords:** 9*H*-9-silafluorene, thiophene, UV absorption, solid-state emission, single-crystal X-ray diffraction, J-aggregation

## Abstract

Aromatic groups were incorporated into 9*H*-9-silafluorene units at the 9-position (mono-9*H*-silafluorenes) and 9,9′-positions (di-9*H*-9-silafluorenes). The aryl substituents showed weak conjugation to the 9*H*-9-silafluorene for 9-aryl substituted ones **1**–**7** and a 9,9′-phenylene substituted one (compound **8**) and they exhibited similar absorption and emission spectra. The 9*H*-9-silafluorene **10** containing a 5,5′-(2,2′-bithiophenyl) group showed a significantly red-shifted absorption and fluorescence maxima in the solid-state. Single-crystal X-ray diffraction studies found J-type aggregated structures formed by intermolecular CH–π interactions (ca. 2.6–2.7 Å). Density functional theory (DFT), time-dependent DFT (TD-DFT), and configuration interaction single (CIS) calculations were conducted to explain the observed optical properties.

## 1. Introduction

9*H*-9-Silafluorenes are attractive building blocks for fluorescent materials because of their appealing optical properties, high stability against light and chemical agents, and high quantum yields [1,2,3,4]. Extending the conjugated π-systems from the 9*H*-9-silafluorene units through the introduction of functional aromatic substituents can shift the absorption/emission maxima to longer wavelengths, and fine-tune the optoelectronic properties. Therefore, the modification and development of 9*H*-9-silafluorenes have been actively investigated. Introducing functional groups on the 9*H*-9-silafluorene ring is an established way to lengthen the π-conjugation system and tune the HOMO–LUMO gaps, and thus essential for the full control of electronic and optical properties [5,6,7,8]. However, despite the interest in 9*H*-9-silafluorenes, their chemistry has not been investigated extensively for the molecular design of fluorescent materials. In particular, there are very few reports on systematic modification with aromatic compounds at the 9-position [9,10].

To obtain emitter molecules with improved photoluminescence properties, we developed a series of 9*H*-9-silafluorene derivatives combined with aromatic rings at the 9-position and investigated their influence on physical properties. The scarcity of prior works reporting functionalization at the 9-position of 9*H*-9-silafluorenes is likely due to the lack of an appropriate synthetic method. Recent developments have suggested that transition-metal-catalyzed arylation of hydrosilane is effective for preparing 9*H*-9-silafluorene-based π-conjugated functional materials, and may facilitate their use as fluorescent materials [11,12,13,14,15]. Our group has been designing and synthesizing silicon-bridged biaryl compounds and investigating their photochemical properties in solution and in the solid state [16,17]. As part of this endeavor, this work reports the synthesis and photophysical properties of a series of 9-aryl-9-methyl-9*H*-9-silafluorenes. Their structure-property relationships are explored through crystallographic analysis and theoretical calculations.

## 2. Results and Discussion

### 2.1. Synthesis and Characterization

The modified 9*H*-9-silafluorene derivatives **1**–**10** (Table 1) were prepared by the Pd-promoted arylation of 9-methyl-9*H*-9-silafluorene [18,19] with aryl iodides in THF using diisopropylethylamine as a base. After an appropriate workup, the derivatives were obtained in up to 56% yield. The reaction worked for both electron-rich (compounds **2**–**5**) and electron-poor aryl iodides (compound **6**). Reactive functional groups, such as an ester substituent, were compatible with the reaction conditions (compound **6**). 1,4-Diiodobenzene, 2,5-diiodothiophene, and 5,5′-diiodo-2,2′-bithiophene were also used in the arylation of 9-methyl-9*H*-9-silafluorene to give 1:2 coupled products **8**–**10**. Compounds **1**, **3** and **5**–**10** were solids and all compounds were soluble in common organic solvents. The resulting chromophores were characterized by ^1^H-NMR, ^13^C{^1^H}-NMR, and HRMS spectroscopies, and were found to be consistent with the proposed structures. Compounds **1**, **3** and **5**–**10** are stable in the solid state, and could be stored without any special precautions.

### 2.2. Absorption and Emission Spectra

Optical properties were investigated by UV-vis and fluorescence spectroscopy. The absorption maxima *λ*_abs_, emission maxima *λ*_em_, fluorescence quantum yields *φ*, and fluorescence lifetimes ***τ*** are listed in Table 2. Figures S1–S4 (see Supplementary Materials) show the compounds′ absorption and emission spectra in degassed *n*-hexane at room temperature. Despite the different aromatic groups at the 9-position, the absorption and emission spectra showed no major differences for **1**–**8** in solution and in the solid state. The electronic absorption spectra contained two peaks at ca. 288 nm and ca. 277 nm, corresponding to the π–π* transition of the 9*H*-9-silafluorene fluorophore. The absorption coefficients of **8**–**10**, which have double 9*H*-9-silafluorene rings, were larger than those of **1**–**7**. The lowest-energy absorptions of **10** showed a bathochromic shift relative to those of **1**–**9**, indicating that the substituent at the 9-position was conjugated with the 5,5′-(2,2′-bithiophenyl) groups. The fluorescence spectra recorded in *n*-hexane showed an emission band at around 340 nm, and appeared similar for all compounds except **10**, whose fluorescence band was red-shifted to 390 nm. The single fluorescence peak suggested that the emission occurred from only the lowest excited state. All the chromophores displayed fluorescence quantum yields of *φ* = ca. 0.1 in *n*-hexane, consistent with values previously reported for 9*H*-9-silafluorenes [20,21,22].

**Figure 1:**
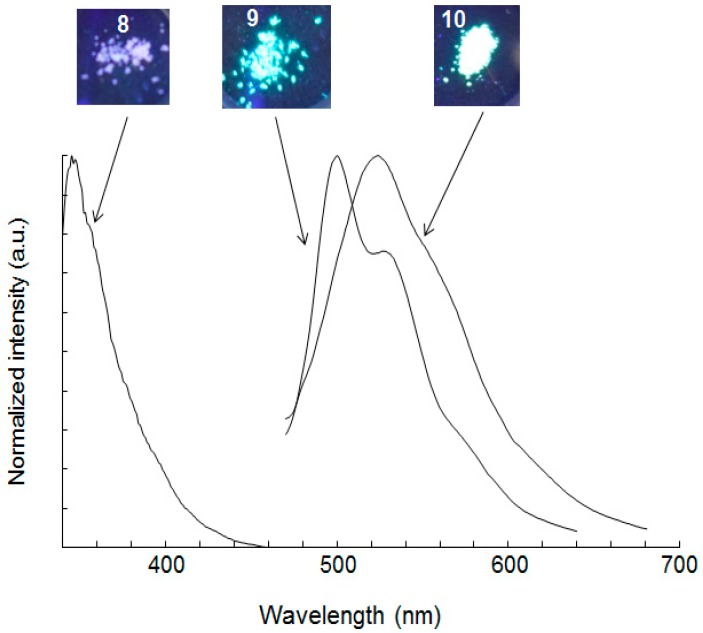
Images of luminescence under UV irradiation and fluorescence spectra of compounds **8**–**10** in the solid state.

Compounds **1**–**8** emitted in the UV-A region with little change in luminescence properties between the solution and the solid state. The nature of the arenyl moiety of the 1:2 coupling products (compounds **9** and **10**) affected the solid state emission considerably (Figure 1). Compounds **1**–**8** emitted in the UV-A range in the solid state, and showed little difference between the solid state and solution spectra like compounds **1**–**7**. The 1:2 coupling products containing a thiophene ring (compound **9**) or a 2,2′-bithiophene ring (compound **10**) had their maximum fluorescence at visible wavelengths (500 nm) with a bathochromic shift of 127 nm relative to the maxima for chromophores **1**–**8**. The fluorescence spectra of **9** (excited at 406 nm) and **10** (excited at 403 nm) in solution displayed red-shifted emission due to the partial solid state-type emission at higher concentration (Figure S5). Fluorescence properties in the solid state are determined by molecular packing in addition to the molecular structure, with aggregation affecting both spectroscopic and photophysical properties. The emissions of **9** and **10** showed slightly higher quantum yield in the solid state than that in solution. They also showed short lifetimes of ca. 0.5 ns, similar to previous reports [23,24]. These results are attributed to the difference in the solid state structure and indicated the formation of J-aggregation. The molecular packing in the solid state was examined using single-crystal X-ray diffraction analysis.

### 2.3. Thermal Stability

To investigate the thermal stability, thermogravimetric and differential thermal analysis (TG-DTA) for **7**–**10** were carried out under a nitrogen atmosphere. Clearly, thermal properties of these compounds were determined by their chemical structures. From the TG-DTA curves shown in Figure S8, mono-9*H*-9-silafluorene **7** remains unchanged upon heating to 200 °C and is decomposed at over 220 °C. On the other hand, aryl-bridged di-9*H*-9-silafluorenes **8** and **9** are thermally stable up to 320 °C and 310 °C (Figures S9 and S10). The highest thermal stability of compound **10** was revealed by the almost constant TG (less than 5% weight loss) up to 360 °C (Figure S11). These results indicated that modified 9*H*-9-silafluorenes displayed high thermal stability, especially aryl-bridged di-9*H*-9-silafluorenes.

### 2.4. X-ray Analysis

**Figure 2:**
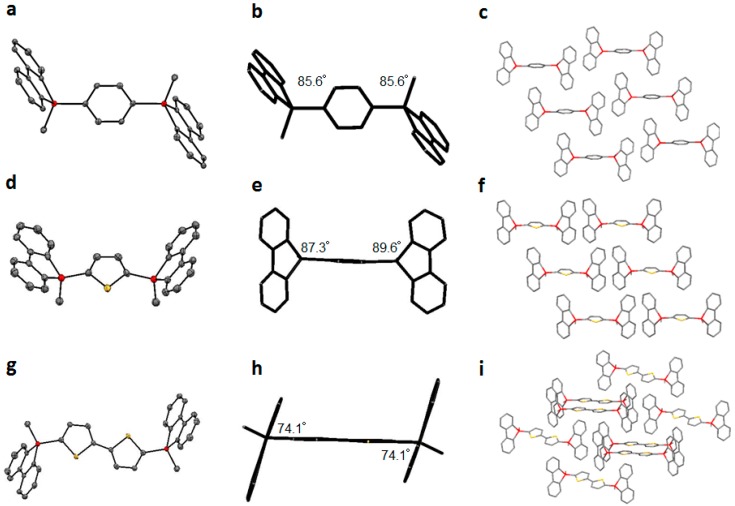
(**a**,**d**,**g**) ORTEP drawings (50% probability ellipsoids), (**b**,**e**,**h**) the angle between siloles and center aryl groups, and (**c**,**f**,**i**) packing structures of (**a**,**b**,**c**) **8**, (**d**,**e**,**f**) **9**, and (**g**,**h**,**i**)**10**. Hydrogen atoms are omitted for clarity.

Single crystals are widely used to study molecular structures, molecular packing, and intermolecular interactions. Single crystals of **8**–**10** were analyzed to explain the red-shifted emissions in crystalline **9** and **10**. The structures and molecular packing were confirmed by X-ray crystallographic analysis (Figure 2) [25].

The Si–C bond lengths were ca. 1.8–1.9 Å, similar to the standard Si–C length previously reported for 9*H*-9-silafluorenes. The 9*H*-9-silafluorene moieties appeared almost flat with deformation of the silicon atom from the ideal geometry of sp^3^ silicon. Compounds **8** and **10** had an *anti* conformation, whereas compound **9** had a *syn* conformation in the solid state. The two thiophenes of **10** were ideally coplanar with a 180° torsion angle, which allowed dense molecular packing. The dihedral angles between the central aryl plane and the 9*H*-9-silafluorene plane are shown in Figure 2. The 9*H*-9-silafluorenes **8** and **9** were positioned almost perpendicular to the central aromatic ring. However, the dihedral angles of **10** between the central bithiophene ring and each 9*H*-9-silafluorene moieties were ca. 74°. These dihedral angles suggest effective σ*–π* conjugation between these planes, especially in compound **9**.

The packing type and interactions strongly affect the solid-state emission. The crystal packing of **8**–**10** had a one-dimensional columnar structure. Their central phenyl or thiophene groups acted on the peripheral 9*H*-9-silafluorene rings by an edge-to-face CH–π interaction with a separation of 2.6–2.7 Å. The shortest distance between the π-planes of adjacent molecules was 6–7 Å. These compounds showed no ring–ring stacking interactions—which cause luminescence quenching—owing to the steric effects of the central aryl group at the 9,9′-position of 9*H*-9-silafluorene.

### 2.5. Theoretical Modeling

**Figure 3:**
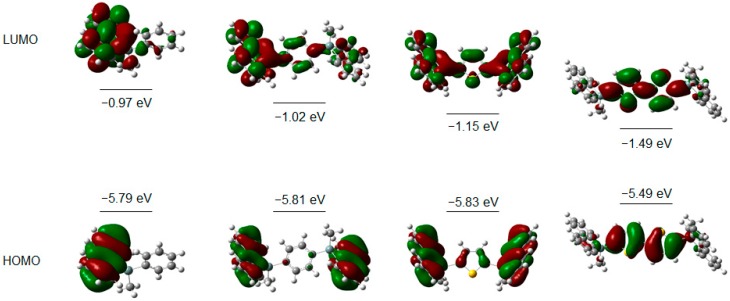
HOMO and LUMO diagrams and calculated energy levels for **1**, **8**, **9**, and **10** (left to right).

To gain more in depth insight into the electronic properties of the target chromophores, DFT, TD-DFT, and CIS calculations were conducted with the Gaussian 09 program using the B3LYP functional and 6-31G* basis set [26]. Figure 3 displays their optimized molecular structures together with the frontier molecular orbital profiles of **1**, **8**, **9**, and **10** as representative compounds. The calculated results are in good agreement with the experimental observation. Figure 3 shows that HOMOs of **1** and **8** are located on the 9*H*-9-silafluorene moiety and no lobe is spread over the phenyl ring at 9- and 9,9′-positions. LUMOs of **1** and **8** are localized on 9*H*-9-silafluorene and silicon bridges. Accordingly, **1** and **8** provided the π–π* transition of 9*H*-9-silafluorene unit in the lowest energy excitation.

In contrast, the LUMO of **9** is spread over the whole molecule and is stabilized by σ*–(Si–C)–π* conjugation in comparison with **1** and **8**. The HOMO and LUMO of **10** are predominantly located on the central 2,2′-bithiophene moiety. The HOMO–LUMO energy gap was decreased in the order of benzene, thiophene, 9*H*-9-silafluorene, and 2,2′-bithiophene. Therefore, the 9*H*-9-silafluorene functionality plays a dominant role in HOMO and LUMO of **1**, **8**, and **9**. The 2,2′-bithiophene moiety in **10** causes the HOMO and LUMO to be localized mainly on the central 2,2′-bithiophene group. Their LUMO energy levels are lower than **1**, whereas the HOMO energies are similar. The energy gap between the HOMO and LUMO gradually decreases in the order of **1** (4.82 eV), **8** (4.79 eV), **9** (4.68 eV), and **10** (4.00 eV). The conjugation of the thiophene groups greatly reduces the HOMO–LUMO energy gap. These calculated results are consistent with the observed bathochromic shift of the lowest-energy absorption wavelength in the UV spectra. The absorption peak of **10** at 323 nm was significantly red-shifted relative to those of the other compounds. The DFT calculations suggest that the longest absorption band of **10** arises from the π–π* transition around the bithiophene ring. The decrease of the HOMO–LUMO energy gap should also affect the emission spectra substantially. The observed spectra suggest that UV-emitting compounds can be tuned with aryl substitution at the 9-position of 9*H*-9-silafluorenes; the subsequent large red shift can lead to a blue–green emission.

Moreover, the dipole moment and transition dipole moment of **8**–**10** were estimated utilizing CIS calculation. The 1,4-phenylene substituent (compound **8**) does not affect the dipole moment (0.05 D) and transition dipole moment (0.08 D), whereas **9** and **10** displayed larger dipole moments (0.52 D for **9** and 0.36 D for **10**) and transition dipole moments (0.25 D for **9** and 10.76 D for **10**), indicative of J-type aggregation in the solid state. The X-ray crystallography results, dipole-dipole interactions, and photophysical properties of **9** and **10** suggested J-aggregation, which accounted for the improved solid-state efficiency and red-shifted fluorescence spectra [27].

## 3. Experimental Section

### 3.1. General Information

All reactions were carried out under an argon atmosphere. All chemicals were purchased from commercial sources and used without further purification unless otherwise noted. 9-Methyl-9*H*-9-silafluorene was synthesized according to the literature [17,18]. Solvents used obtained from a solvent purification system. Melting points were measured with a Yanaco Micro Melting Point apparatus (Yanaco, Tokyo, Japan) and are uncorrected. ^1^H- and ^13^C{^1^H}-NMR spectra were recorded on a US-500 spectrometer (Bruker corporation, Billerica, MA, USA) at 500 and 125 MHz, respectively. GC-MS spectra were recorded with a GC-MS-QP2010 spectrometer (Shimadzu, Tokyo, Japan). FAB mass spectra were measured with a MStation JMS-700 spectrometer (JEOL Ltd., Tokyo, Japan). Absorption spectra were measured with a JASCO V-570 spectrometer (JASCO, Tokyo, Japan). Fluorescence spectra were measured with a F-4500 spectrometer (Hitachi, Tokyo, Japan). Fluorescent quantum yields were recorded with a C9920-02 spectrometer (Hamamatsu, Shizuoka, Japan) and determined with an integrating sphere. Fluorescence lifetimes were measured with a Hamamatsu C11367 spectrometer (Hamamatsu, Shizuoka, Japan). Preparative gel permeation chromatographic separation was carried out with a LC-9110 NEXT recycling preparative HPLC system (JAI, Tokyo, Japan). Thermogravimetric and differential thermal analysis (TG-DTA) were measured with a Thermoplus TG 8120 (Rigaku, Tokyo, Japan). All TG-DTA measurements were conducted under N_2_ atmosphere with heating rate as 10 °C/min and Al_2_O_3_ was used for reference. All calculations were performed at the B3LYP/6-31G* level to extract theoretical electronic properties and correlated with experimental trends.

### 3.2. Typical Experimental Procedure for the Preparation of ***1**–**7***

Iodoarene (1.0 mmol), 9-methyl-9*H*-9-silafluorene (2.0 mmol), and triethylamine (3.0 mmol) were added to a solution of Pd(P(*t*-Bu)_3_)_2_ (0.05 mmol) in THF (1.0 M). The reaction mixture was stirred at room temperature for 1 day under argon, and then quenched with water. The aqueous layer was extracted with dichloromethane three times and dried over sodium sulfate. The solvent was removed in vacuo, and the residue was purified by column chromatography to afford the desired mono arylated 9*H*-9-silafluorenes. Analytically pure compounds were isolated by purification with GPC or recrystallization.

### 3.3. Typical Experimental Procedure for the Preparation of ***8**–**10***

Diiodoarene (2.0 mmol), 9-methyl-9*H*-9-silafluorene (1.0 mmol), and triethylamine (6.0 mmol) were added to a solution of Pd(P(*t*-Bu)_3_)_2_ (0.05 mmol) in THF (1.0 M). The reaction mixture was stirred at room temperature for 1 day under argon, and then quenched with water. The aqueous layer was extracted with dichloromethane three times and dried over sodium sulfate. The solvent was removed in vacuo, and the residue was purified by column chromatography to afford the desired di-arylated 9*H*-9-silafluorenes. Analytically pure compounds were isolated by purification with GPC or recrystallization.

### 3.4. X-ray Crystallography

X-ray diffraction data for **8** were collected at 93 K on a Rigaku Saturn 724 (Rigaku, Tokyo, Japan) diffractometer with multi-layer mirror monochromated Mo Kα radiation (*λ* = 0.71075 Å). X-ray diffraction data of **9** and **10** were collected at 113 K with an AFC10 (Rigaku, Tokyo, Japan) diffractometer coupled with a Rigaku Saturn CCD system equipped with a rotating-anode X-ray generator producing graphite monochromated Mo Kα (*λ* = 0.71070 Å) radiation. Lorentz-polarization and empirical absorption corrections were performed with the program Crystal Clear 2.0 (Rigaku, Tokyo, Japan) (**8**) or Crystal Clear 1.3.6 (Rigaku, Tokyo, Japan) (**9** and **10**). The structures were solved by the direct method using SIR-92 program [28], and refined by the full-matrix least-squares techniques against *F*^2^ implementing SHELXL-97 [29,30].

### 3.5. Physical Data of ***1**–**10***

*9-Methyl-9-phenyl-9H-9-silafluorene* (**1**) [18,19]. Yield: 41%. Colorless solid. ^1^H-NMR (CDCl_3_) δ 7.86 (d, 2H, *J* = 7.6 Hz), 7.65 (d, 2H, *J* = 6.9 Hz), 7.55 (d, 2H, *J* = 6.7 Hz), 7.45 (t, 2H, *J* = 7.7 Hz), 7.38–7.26 (m, 5H), 0.73 (s, 3H). ^13^C{^1^H}-NMR (CDCl_3_) δ 148.4 (C_q_), 137.4 (C_q_), 134.6 (C_q_), 134.5 (CH), 133.4 (CH), 130.5 (CH), 129.8 (CH), 128.1 (CH), 127.6 (CH), 121.0 (CH), −5.0 (CH_3_). EI-MS *m*/*z* 272 [M]^+^.

*9-Methyl-9-(p-tolyl)-9H-9-silafluorene* (**2**). Yield: 24%. Colorless oil. ^1^H-NMR (CDCl_3_) δ 7.85 (d, 2H, *J* = 7.6 Hz), 7.63 (dd, 2H, *J* = 6.3 Hz, 3.0 Hz), 7.44–7.42 (m, 3H), 7.27 (td, 2H, *J* = 7.3 Hz, 0.6 Hz), 7.13 (d, 2H, *J* = 7.6 Hz), 2.34 (s, 3H), 0.71 (s, 3H). ^13^C{^1^H}-NMR (CDCl_3_) δ 148.4 (C_q_), 139.9 (C_q_), 137.7 (C_q_), 134.6 (CH), 133.4 (CH), 130.9 (C_q_), 130.4 (CH), 128.9 (CH), 127.6 (CH), 121.0 (CH), 21.6 (CH_3_), −5.0 (CH_3_). EI-MS *m*/*z* 286 [M]^+^. HRMS (FAB) *m*/*z* [M + H]^+^ Calcd for C_20_H_19_Si 287.1256; found 287.1241.

*9-Methyl-9-(m-tolyl)-9H-9-silafluorene* (**3**). Yield: 33%. Colorless solid. Mp: 108.8–110.3 °C. ^1^H-NMR (CDCl_3_) δ 7.87 (d, 2H, *J* = 7.6 Hz), 7.68–7.64 (m, 3H), 7.49 (td, 2H, *J* = 7.6 Hz, 1.3 Hz), 7.31–7.25 (m, 3H), 7.18 (t, 1H, *J* = 7.4 Hz), 7.11 (d, 1H, *J* = 7.3 Hz), 2.15 (s, 3H), 0.73 (s, 3H). ^13^C{^1^H}-NMR (CDCl_3_) δ 148.1 (C_q_), 144.9 (C_q_), 137.9 (C_q_), 136.0 (CH), 133.4 (CH), 132.9 (C_q_), 130.3 (CH), 130.2 (CH), 129.8 (CH), 127.6 (CH), 125.1 (CH), 121.1 (CH), 22.8 (CH_3_), −3.6 (CH_3_). EI-MS *m*/*z* 286 [M]^+^. HRMS (FAB) *m*/*z* [M + H]^+^ Calcd for C_20_H_19_Si 287.1256; found 287.1241.

*9-Methyl-9-(o-tolyl)-9H-9-silafluorene* (**4**). Yield: 56%. Colorless oil. ^1^H-NMR (CDCl_3_) δ 7.86 (d, 2H, *J* = 7.9 Hz), 7.65 (d, 2H, *J* = 7.6 Hz), 7.45 (td, 2H, *J* = 7.6 Hz, 1.3 Hz), 7.36 (m, 2H), 7.27 (td, 2H, *J* = 7.3 Hz, 0.9 Hz), 7.23–7.17 (m, 2H), 2.29 (s, 3H), 0.72 (s, 3H). ^13^C{^1^H}-NMR (CDCl_3_) δ 148.4 (C_q_), 137.6 (C_q_), 137.5 (C_q_), 135.0 (CH), 134.4 (C_q_), 133.4 (CH), 131.6 (CH), 130.8 (CH), 130.5 (CH), 128.0 (CH), 127.6 (CH), 121.0 (CH), 21.5 (CH_3_), −4.9 (CH_3_). EI-MS *m*/*z* 286 [M]^+^. HRMS (FAB) *m*/*z* [M + H]^+^ Calcd for C_20_H_19_Si 287.1256; found 287.1241.

*9-(p-Methoxyphenyl)-9-methyl-9H-9-silafluorene* (**5**). Yield 39%. Colorless solid. Mp: 88.5–90.0 °C. ^1^H-NMR (CDCl_3_) δ 7.86 (d, 2H, *J* = 7.6 Hz), 7.62 (d, 2H, *J* = 7.6 Hz), 7.48–7.42 (m, 4H), 7.26 (t, 2H, *J* = 6.8 Hz), 6.87 (d, 2H, *J* = 8.8 Hz), 3.77 (s, 3H), 0.70 (s, 3H). ^13^C{^1^H}-NMR (CDCl_3_) δ 161.2 (C_q_), 148.3 (C_q_), 137.8 (C_q_), 136.0 (CH), 133.3 (CH), 130.4 (CH), 127.6 (CH), 125.2 (C_q_), 120.9 (CH), 113.9 (CH), 55.1 (CH_3_), −4.9 (CH_3_). EI-MS *m*/*z* 302 [M]^+^. HRMS (FAB) *m*/*z* [M]^+^ Calcd for C_20_H_18_OSi 302.1127; found 302.1115.

*Ethyl 4-(9-methyl-9H-9-silafluoren-9-yl)benzoate* (**6**). Yield: 28%. Colorless solid. Mp: 101.3–102.5 °C. ^1^H-NMR (CDCl_3_) δ 7.97 (d, 2H, *J* = 8.2 Hz), 7.87 (d, 2H, *J* = 7.7 Hz), 7.64–7.61 (m, 4H), 7.47 (td, 2H, *J* = 7.6 Hz, 1.3 Hz), 7.29 (t, 2H, *J* = 7.1 Hz), 4.35 (q, 2H, *J* = 8.3 Hz), 1.36 (t, 3H, *J* = 7.1 Hz), 0.76 (s, 3H). ^13^C{^1^H}-NMR (CDCl_3_) δ 166.6 (C_q_), 148.5 (C_q_), 140.9 (C_q_), 136.7 (C_q_), 134.4 (CH), 133.4(CH), 131.6 (C_q_), 130.8 (CH), 128.8 (CH), 127.8 (CH), 121.1 (CH), 61.0 (CH_2_), 14.3 (CH_3_), -5.2 (CH_3_). EI-MS *m/z* 344 [M]^+^. HRMS (FAB) *m/z* [M]^+^ Calcd for C_22_H_20_O_2_Si 344.1236; found 344.1210.

*9-Methyl-9-(thiophen-2-yl)-9H-9-silafluorene* (**7**). Yield: 37%. Colorless solid. Mp: 70.3–71.5 °C. ^1^H-NMR (CDCl_3_) δ 7.84 (d, 2H, *J* = 7.9 Hz), 7.67 (d, 2H, *J* = 7.2 Hz), 7.62 (dd, 1H, *J* = 4.7 Hz, 1.0 Hz), 7.46 (td, 2H, *J* = 7.5 Hz, 1.4 Hz), 7.34 (dd, 1H, *J* = 3.5 Hz, 1.0 Hz), 7.28 (td, 2H, *J* = 7.3 Hz, 1.0 Hz), 7.17 (dd, 1H, *J* = 4.6 Hz, 3.2 Hz), 0.77 (s, 3H). ^13^C{^1^H}-NMR (CDCl_3_) δ 148.1 (C_q_), 136.8 (C_q_), 136.4 (CH), 133.4 (C_q_), 133.3 (CH), 132.1 (CH), 130.8 (CH), 128.4 (CH), 127.7 (CH), 121.0 (CH), −3.6 (CH_3_). EI-MS *m*/*z* 278 [M]^+^. HRMS (FAB) *m*/*z* [M]^+^ Calcd for C_17_H_14_SSi 278.0585; found 278.0570.

*1,4-Bis(9-methyl-9H-9-silafluoren-9-yl)benzene* (**8**). Yield: 11%. Colorless solid. Mp: 193.2–194.5 °C. ^1^H-NMR (CDCl_3_) δ 7.83 (d, 4H, *J* = 7.6 Hz), 7.59 (d, 4H, *J* = 6.6 Hz), 7.49 (s, 4H), 7.43 (td, 4H, *J* = 7.4 Hz, 1.2 Hz), 7.24 (td, 4H, *J* = 7.1 Hz, 0.8 Hz), 0.69 (s, 6H). ^13^C{^1^H}-NMR (CDCl_3_) δ 148.4 (C_q_), 137.1 (C_q_), 136.5 (C_q_), 133.9 (CH), 133.3 (CH), 130.5 (CH), 127.6 (CH), 121.0 (CH), −5.3 (CH_3_). EI-MS *m*/*z* 466 [M]^+^. HRMS (FAB) *m*/*z* [M]^+^ Calcd for C_32_H_26_Si_2_ 466.1573; found 466.1591.

*2,5-Bis(9-methyl-9H-9-silafluoren-9-yl)thiophene* (**9**). Yield: 13%. Pale yellow solid. Mp: 179.5–181.0 °C. ^1^H-NMR (CDCl_3_) δ 7.83 (d, 4H, *J* = 7.6 Hz), 7.64 (d, 4H, *J* = 7.0 Hz), 7.44 (t, 4H, *J* = 7.0 Hz), 7.36 (s, 2H), 7.27 (t, 4H, *J* = 7.3 Hz), 0.7 (s, 6H). ^13^C{^1^H}-NMR (CDCl_3_) δ 148.1 (C_q_), 141.1 (C_q_), 137.6 (CH), 136.7 (C_q_), 133.4 (CH), 130.8 (CH), 127.8 (CH), 121.1 (CH), −3.5 (CH_3_). EI-MS *m*/*z* 472 [M]^+^. HRMS (FAB) *m*/*z* [M]^+^ Calcd for C_30_H_24_SSi_2_ 472.1137; found 472.1147.

*5,5′-Bis(9-methyl-9H-9-silafluoren-9-yl)-2,2′-bithiophene* (**10**). Yield: 17%. Yellow solid. Mp: 251.2–252.8 °C. ^1^H-NMR (CDCl_3_) δ 7.84 (d, 4H, *J* = 7.6 Hz), 7.66 (d, 4H, *J* = 6.7 Hz), 7.46 (td, 4H, *J* = 7.6 Hz, 1.3 Hz), 7.28 (td, 4H, *J* = 7.6 Hz, 0.7 Hz), 7.18 (d, 2H, *J* = 3.5 Hz), 7.17 (d, 2H, *J* = 3.5 Hz), 0.75 (s, 6H). ^13^C{^1^H}-NMR (CDCl_3_) δ 148.1 (C_q_), 143.7 (C_q_), 137.2 (CH), 136.4 (C_q_), 133.3 (CH), 130.9 (CH), 127.8 (CH), 125.6 (CH), 121.0 (CH), −3.9 (CH_3_). EI-MS *m*/*z* 554 [M]^+^. HRMS (FAB) *m*/*z* [M]^+^ Calcd for C_34_H_26_S_2_Si_2_ 554.1015; found 554.0992.

## 4. Conclusions

We have designed and synthesized 9*H*-9-silafluorene derivatives with different aryl groups incorporated at the 9-position. These fluorophores were formed through Pd-catalyzed arylation of 9*H*-9-silafluorene and aryl iodides. Various aromatic substituents at the 9-position were compared. Compounds **1**–**8**, with various substituents on the silicon atom, all showed similar UV–Vis and emission spectra. Compounds **9** and **10** showed different solid-state emissions when aggregated owing to the modulation of their intermolecular interactions. The fluorescence of **9** and **10** was characterized by a large Stokes shift. The thermal stabilities of **7**–**10** were investigated by TG-DTA measurement. These compounds remain unchanged upon heating to 200 °C (compound **7**), 320 °C (compound **8**), 310 °C (compound **9**), and 360 °C (compound **10**), respectively. Theoretical calculations showed that thiophenyl or bithiophenyl substitutions of 9*H*-9-silafluorene at the 9,9′-positions decreased the HOMO–LUMO energy gap. Consistent with the calculations, these two compounds showed red-shifted UV–vis and emission spectra compared with the other compounds.

The solid-state optical and photophysical properties of the compounds were primarily affected by their molecular packing and dipole-dipole interaction. Our method facilitated the J-aggregated formation of fluorophores via a simple chemical-group substitution. Intermolecular CH–π interactions in the crystal assisted the slipped–stacked arrangement, and strongly affected the solid-state emission. The hindrance of the rotation in the solid state yielded higher quantum yields. Given that the substitution can be easily achieved by a Pd-catalyzed coupling reaction, this synthetic approach appears useful to extend and improve these molecules’ fluorescent properties.

## Figures and Tables

**Table 1:**
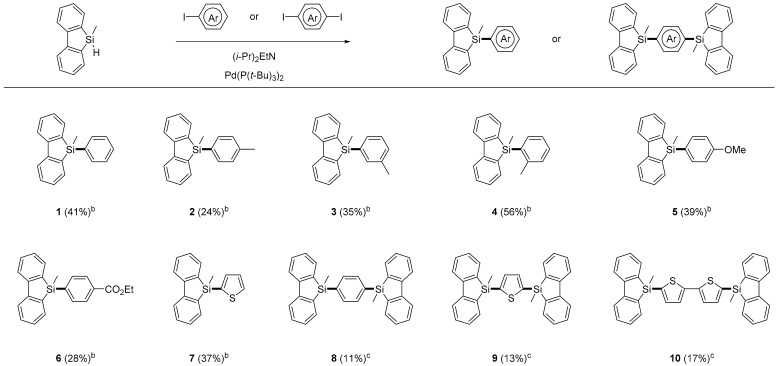
Structures and yields of 9*H*-9-silafluorenes **1**–**10** ^a^.

^a^ Numbers in parentheses are isolated yield. *Reagents and* *Conditions*: ^b^ Iodoarene (1.0 mmol), 9-methyl-9*H*-9-silafluorene (2.0 mmol), and triethylamine (3.0 mmol), Pd(P(*t*-Bu)_3_)_2_ (0.05 mmol), THF (1.0 M), 1 day, under Ar. ^c^ Iodoarene (2.0 mmol), 9-methyl-9*H*-9-silafluorene (1.0 mmol), and triethylamine (6.0 mmol), Pd(P(*t*-Bu)_3_)_2_ (0.05 mmol), THF (1.0 M), 1 day, under Ar.

**Table 2:** Photophysical properties of 9*H*-9-silafluorene derivatives **1**–**10** ^a^.

Compound	In *n*-Hexane					In the Solid State			
	*λ*_abs_ (nm) ^b^	ε (10^4^ M^−1^·cm^−1^)	*λ*_em_ (nm) ^c^	*Φ* ^d^	*τ* (ns) ^e^	*λ*_ex_ (nm)	*λ*_em_ (nm) ^c^	*Φ* ^d^	*τ* (ns)
**1**	277, 287	1.10	342	0.08	4.0	320	346	0.05	7.2 ^e^
**2**	277, 288	1.10	340	0.10	3.9	- ^f^	- ^f^	- ^f^	- ^f^
**3**	278, 289	1.73	341	0.07	3.7	324	345	0.18	7.2^e^
**4**	278, 289	1.39	341	0.14	4.0	- ^f^	- ^f^	- ^f^	- ^f^
**5**	280, 288	1.66	340	0.04	3.8	321	344	0.18	7.8 ^e^
**6**	277, 288	1.24	343	0.09	3.9	326	348	0.18	7.7 ^e^
**7**	277, 288	1.16	344	0.07	3.6	323	347, 385	0.11	3.3, 0.9 ^e^
**8**	277, 289	2.25	342	0.12	3.8	321	345	0.11	4.4 ^e^
**9**	264, 277, 289	3.31	348, 490 ^g^	0.07	2.3	444	500	0.20	0.5 ^h^
**10**	279, 323	2.54	390, 505 ^i^	0.04	0.6	446	524	0.06	0.6 ^h^

^a^ All measurements were carried out at rt. ^b^ Maximum absorption wavelength. Underlined values are wavelengths with the highest peak intensity. ^c^ Maximum fluorescence wavelength at *λ*_abs_ or *λ*_ex_. ^d^ Absolute fluorescent quantum yield measured with an integrating sphere. ^e^ Fluorescence lifetime measured by photoexcitation at 280 nm. ^f^ Compounds obtained as oils. ^g^ Excited at 406 nm. ^h^ Fluorescence lifetime measured by photoexcitation at 406 nm. ^i^ Excited at 403 nm.

## Supplementary Materials

Supplementary materials are available online at http://www.mdpi.com/1420-3049/21/9/1173/s1.
