# Supplementary material for: Effects of Substitution on Solid-State Fluorescence in 9-Aryl-9-methyl-9H-9-silafluorenes
Source: Molecules. 2016 Sep 3;21(9):1173. doi: 10.3390/molecules21091173 (PMC6274553; doi:10.3390/molecules21091173)
Supplement: Supplementary file 1 [file molecules-21-01173-s001.pdf]

Supplementary Materials: Effects of Substitution on Solid-State Fluorescence in 9-Aryl-9-methyl-9*H*-9-silafluorenes

Yoshinori Yamanoi, Takayuki Nakashima, Masaki Shimada, Hiroaki Maeda and Hiroshi Nishihara

Contents

1. Absorption spectra of **1–10** in *n*-hexane S2
2. Fluorescence spectra of **1–10** in *n*-hexane S2
3. Fluorescence spectra of **9** and **10** under various conditions S3
4. Fluorescence spectra of **1**, **3**, and **5–7** in the solid state S4
5. TG-DTA measurement of **7–10** S4
6. Crystallographic data of **8–10** S6
7. Copies of ^{1}H-NMR and ^{13}C{^{1}H}-NMR of **1–10** S9

1. Absorption Spectra of 1–10 in *n*-Hexane

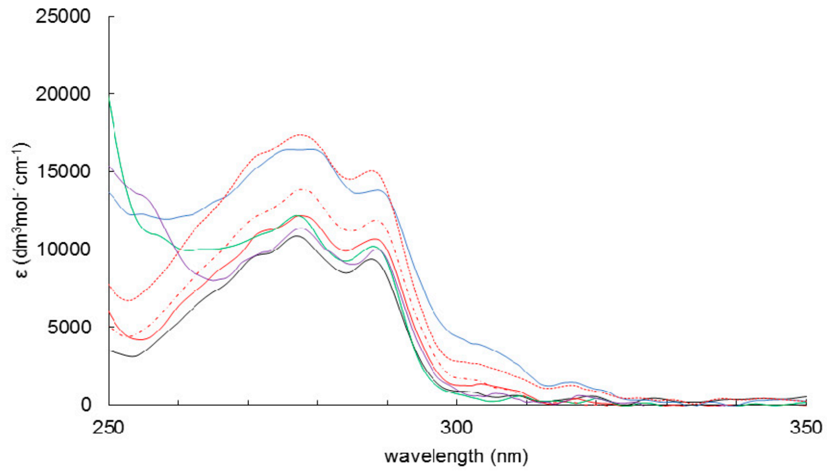

Figure S1. Absorption spectra of **1–7** in *n*-hexane.

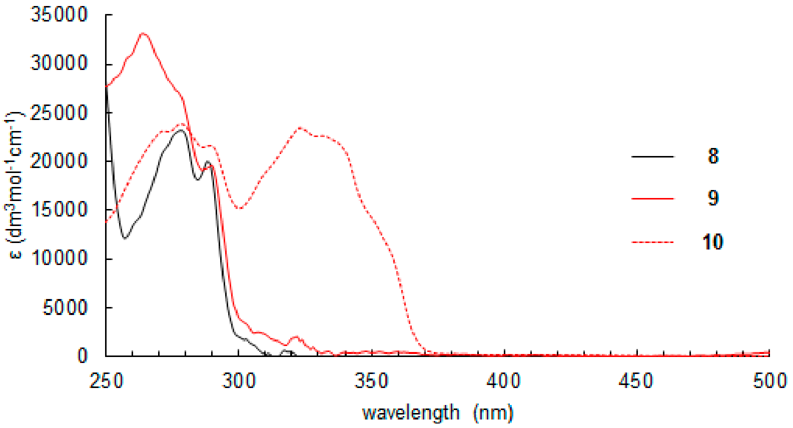

Figure S2. Absorption spectra of **8–10** in *n*-hexane.

2. Fluorescence Spectra of 1–10 in *n*-Hexane

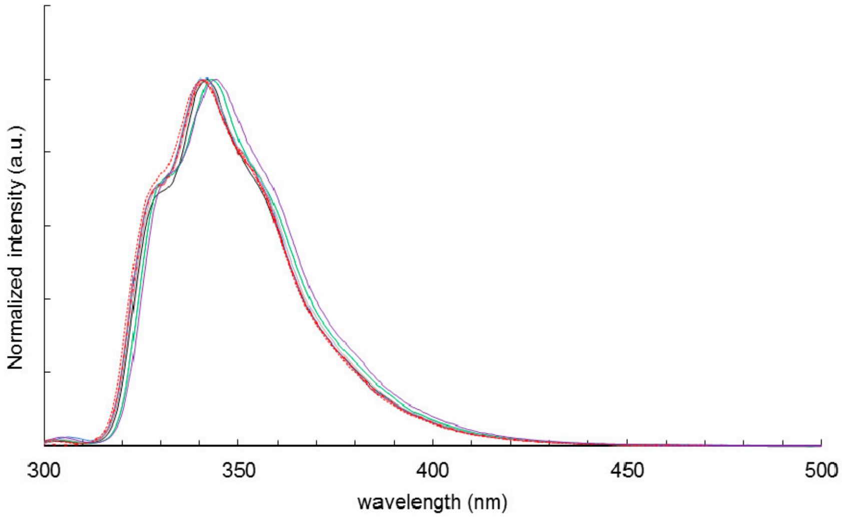

Figure S3. Fluorescence spectra of **1–7** in *n*-hexane.

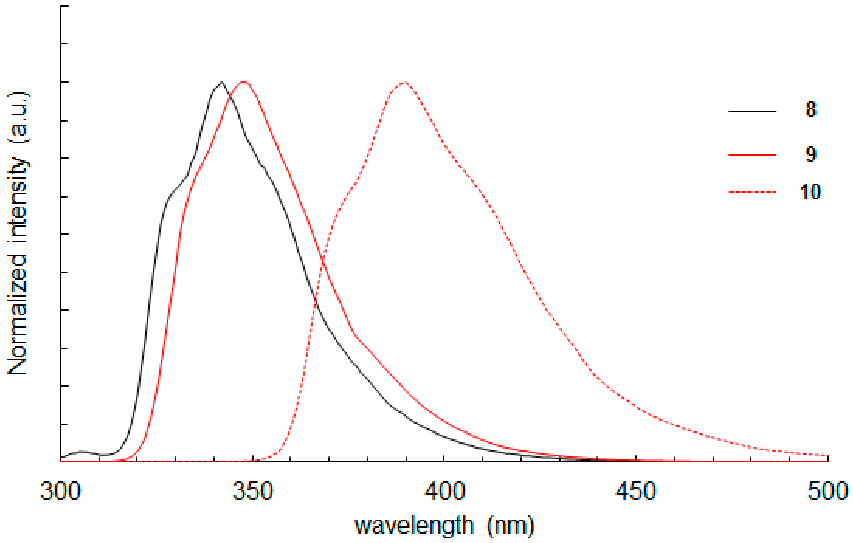

Figure S4. Fluorescence spectra of **8–10** in *n*-hexane.

3. Fluorescence Spectra of 9 and 10 under Various Conditions

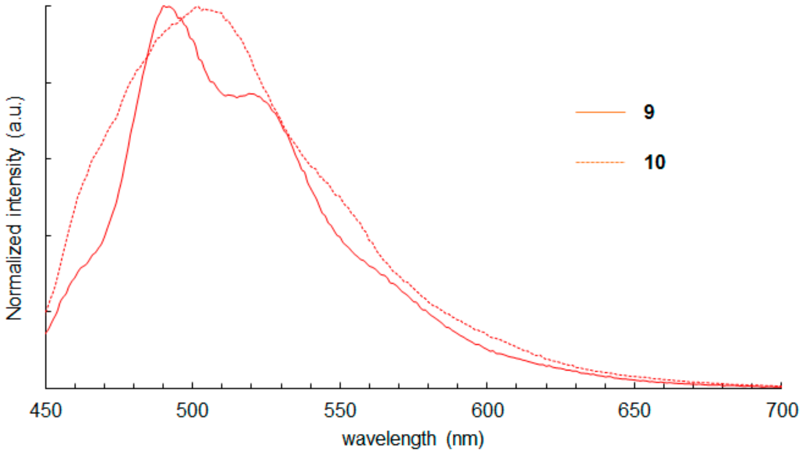

Figure S5. Fluorescence spectra of **9** (excited at 406 nm) and **10** (excited at 402 nm) in *n*-hexane.

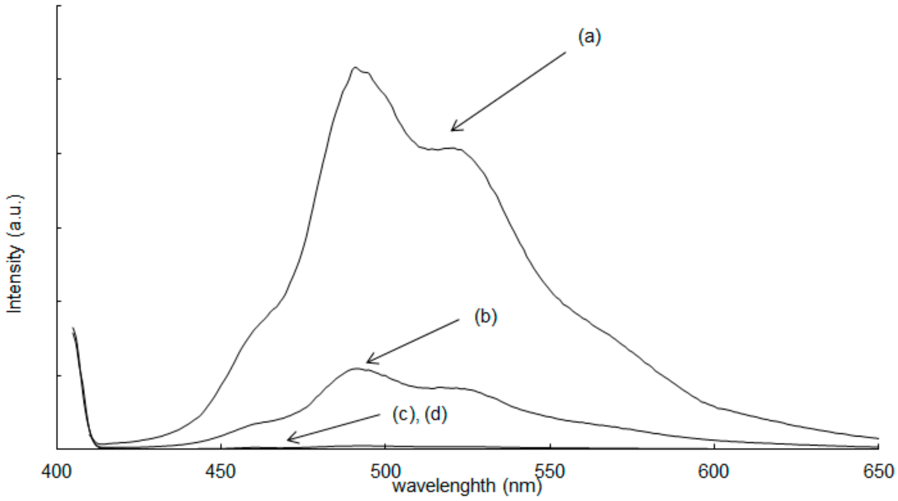

Figure S6. Fluorescence spectra of **9** (excited at 406 nm) in *n*-hexane under (a) 10^{-3} M, (b) 10^{-4} M, (c) 10^{-5} M and (d) 10^{-6} M.

4. Fluorescence Spectra of 1, 3, and 5–7 in the Solid State

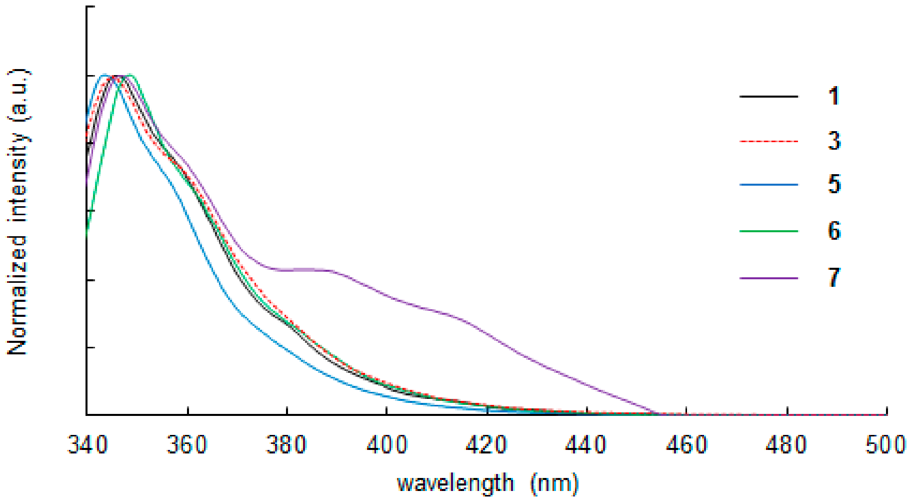

Figure S7. Fluorescence spectra of **1**, **3**, and **5**–**7** in the solid state.

5. TG-DTA Measurement of 7–10

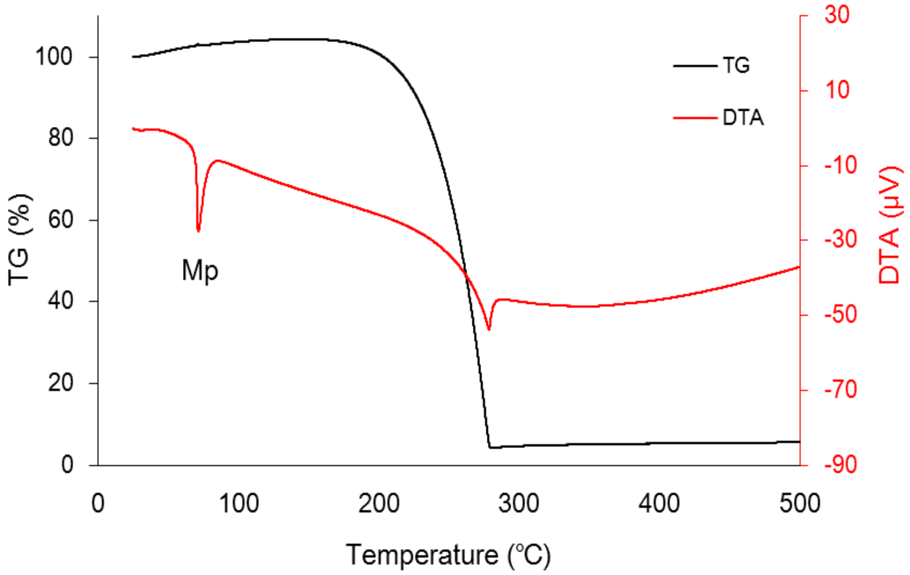

Figure S8. TG-DTA spectra of **7**.

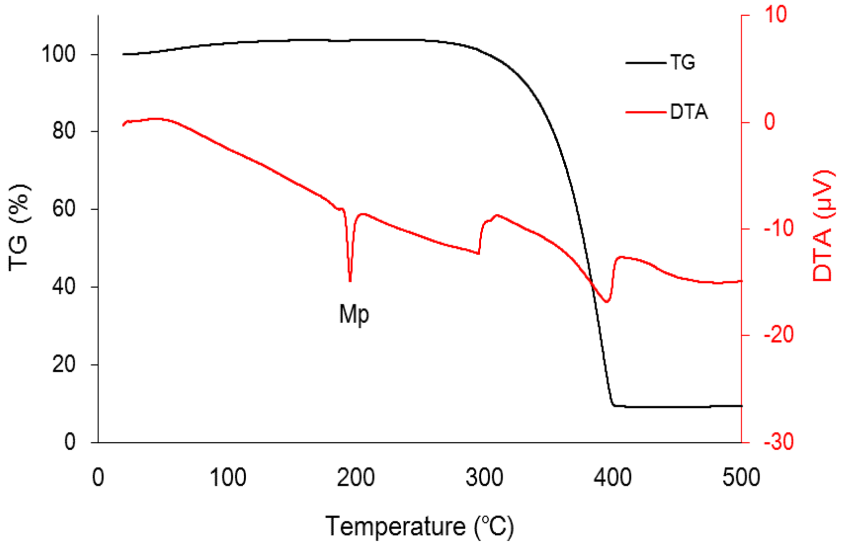

Figure S9. TG-DTA spectra of **8**.

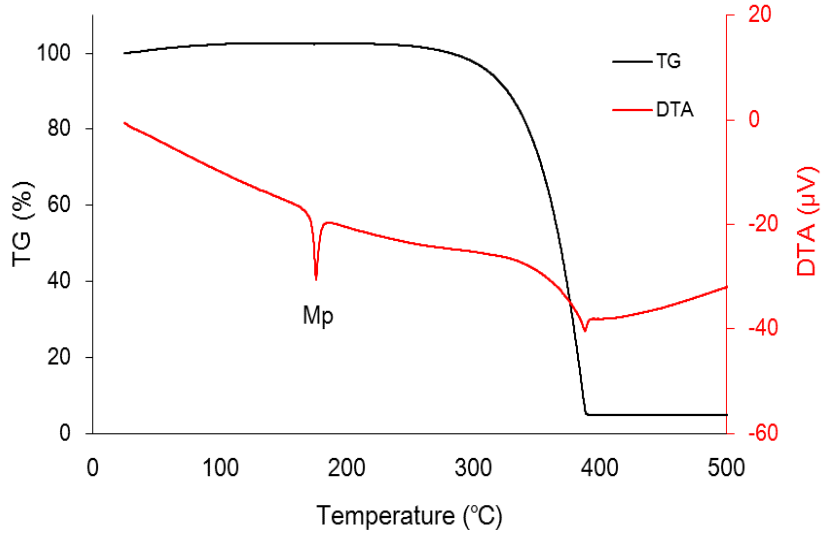

Figure S10. TG-DTA spectra of **9**.

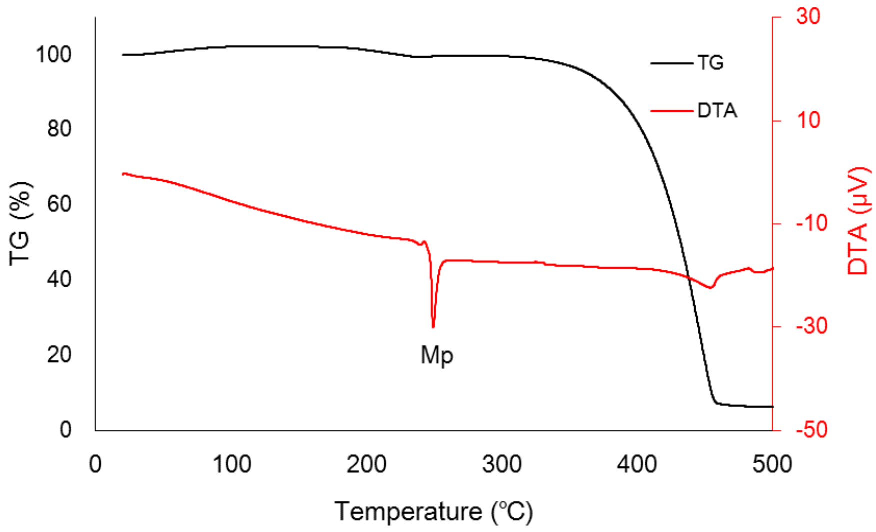

Figure S11. TG-DTA spectra of **10**.

6. Crystallographic Data of 8–10

Table S1. Selected crystallographic data of **8**.

**Empirical Formula**	$C_{32}H_{26}Si_2$
F_w/g·mol^{-1}	466.73
Crystal system	monoclinic
Space group	$P2_1/c$
Crystal size/mm	0.250 × 0.170 × 0.060
Temperature/K	93
a/Å	13.373(7)
b/Å	11.094(5)
c/Å	8.642(4)
α/°	90
β/°	104.726(6)
γ/°	90
V/Å^3	1240.0(11)
Z	2
D_{calcd}/g·cm^{-3}	1.250
λ/Å	0.71075
μ/mm^{-1}	0.1618
Reflections collected	8732
Independent reflections	2504
Parameters	154
R_{int}	0.0480
R_1 (I >2.00σ (I)) [a]	0.0486
wR_2 (All reflections) [b]	0.1053
GoF [c]	1.119

[a] $R_1 = \Sigma||F_o| - |F_c||/\Sigma|F_o|$ ($I > 2\sigma(I)$). [b] $wR_2 = [\Sigma(w(F_o^2 - F_c^2)^2/\Sigma w(F_o^2)^2]^{1/2}$ ($I > 2\sigma(I)$). [c] GoF = $[\Sigma(w(F_o^2 - F_c^2)^2/\Sigma(N_r - N_p)^2]$.

Table S2. Selected bond lengths, angles, and torsion angles of **8**.

**Bond lengths (Å)**	
Si1–C2	1.865 (3)
Si1–C3	1.873 (3)
Si1–C8	1.8713 (19)
Si1–C13	1.861 (3)
**Angles (°)**	
C2–Si1–C3	91.55 (9)
C2–Si1–C8	112.02 (10)
C2–Si1–C13	113.78 (10)
C3–Si1–C8	115.66 (10)
C3–Si1–C13	112.05 (10)
C8–Si1–C13	110.66 (9)

Table S3. Selected crystallographic data of **9**.

Empirical Formula	$C_{30}H_{24}SSi_2$
F_w/g·mol^{-1}	472.75
Crystal system	triclinic
Space group	*P*-1
Crystal size/mm	0.110 × 0.061 × 0.030
Temperature/K	113
a/Å	8.717(3)
b/Å	12.385(4)
c/Å	12.634(4)
α/°	81.455(13)
β/°	76.448(12)
γ/°	69.996(10)
V/Å^3	1242.5(7)
Z	2
D_{calcd}/g·cm^{-3}	1.264
λ/Å	0.71070
μ/mm^{-1}	0.243
Reflections collected	9020
Independent reflections	4770
Parameters	298
R_{int}	0.0335
R_1 (I >2.00$\sigma(I)$) [a]	0.0616
wR_2 (All reflections) [b]	0.1634
GoF [c]	1.070

[a] $R_1 = \Sigma||Fo| - |Fc||/\Sigma|Fo|$ ($I > 2\sigma(I)$). [b] $wR_2 = [\Sigma(w(Fo^2 - Fc^2)^2/\Sigma w(Fo^2)^2]^{1/2}$ ($I > 2\sigma(I)$). [c] GoF = $[\Sigma(w(Fo^2 - Fc^2)^2/\Sigma(Nr - Np)^2]$.

Table S4. Selected bond lengths, angles, and torsion angles of **9**.

**Bond lengths (Å)**	
Si2–C4	1.879(4)
Si2–C15	1.873(4)
Si2–C16	1.863(4)
Si2–C17	1.863(3)
Si3–C20	1.867(3)
Si3–C21	1.863(4)
Si3–C32	1.866(4)
Si3–C33	1.855(4)
**Angles (°)**	
C4–Si2–C15	91.53(16)
C4–Si2–C16	112.69(17)
C4–Si2–C17	113.69(17)
C15–Si2–C16	114.32(17)
C15–Si2–C17	114.98(14)
C16–Si2–C17	108.88(15)
C20–Si3–C21	112.45(17)
C20–Si3–C32	111.40(14)
C20–Si3–C33	109.03(15)
C21–Si3–C32	91.95(16)
C21–Si3–C33	114.85(16)
C32–Si3–C33	116.39(17)

Table S5. Selected crystallographic data of **10**.

Empirical Formula	$C_{34}H_{26}S_2Si_2$
F_w/g·mol^{-1}	554.87
Crystal system	monoclinic
Space group	$P2_1/c$
Crystal size/mm	0.260 × 0.218 × 0.202
Temperature/K	113
a/Å	10.972(4)
b/Å	6.584(2)
c/Å	20.551(7)
α/°	90
β/°	102.513(4)
γ/°	90
V/Å^3	1449.2(8)
Z	2
D_{calcd}/g·cm^{-3}	1.271
λ/Å	0.71070
μ/mm^{-1}	0.288
Reflections collected	10960
Independent reflections	3217
Parameters	172
R_{int}	0.0534
R_1 (I >2.00σ(I)) [a]	0.0540
wR_2 (All reflections) [b]	0.1180
GoF [c]	1.094

[a] $R_1 = \Sigma||Fo| - |Fc||/\Sigma|Fo|$ ($I > 2\sigma$ (I)). [b] $wR_2 = [\Sigma(w(Fo^2 - Fc^2)^2/\Sigma w(Fo^2)^2]^{1/2}$ ($I > 2\sigma$ (I)). [c] GoF = $[\Sigma(w(Fo^2 - Fc^2)^2/\Sigma(Nr - Np)^2]$.

Table S6. Selected bond lengths, angles, and torsion angles of **10**.

**Bond lengths (Å)**	
Si2–C6	1.859(3)
Si2–C7	1.860(3)
Si2–C8	1.869(3)
Si2–C19	1.869(3)
**Angles (°)**	
C6–Si2–C7	109.10(12)
C3–Si2–C8	114.72(11)
C6–Si2–C19	111.57(11)
C7–Si2–C8	112.77(12)
C7–Si2–C19	116.28(11)

7. Copies of ^{1}H-NMR and ^{13}C{^{1}H} NMR of 1–10

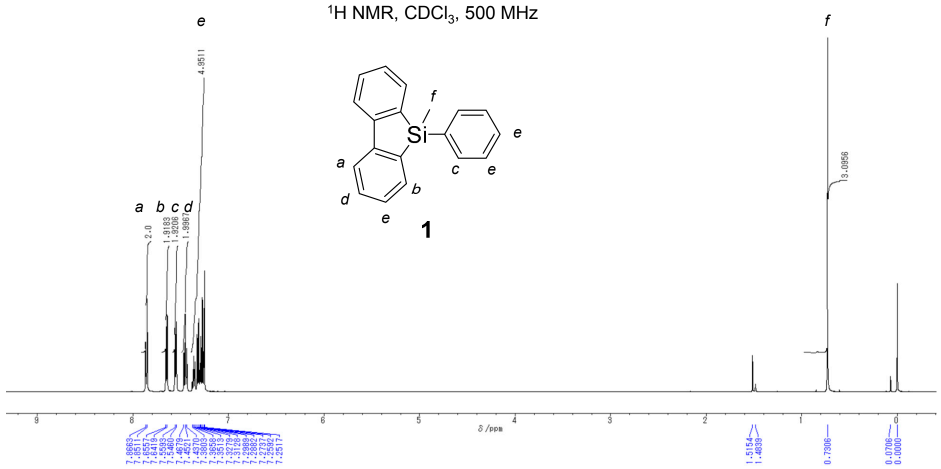

Figure S12. ^{1}H-NMR of **1**.

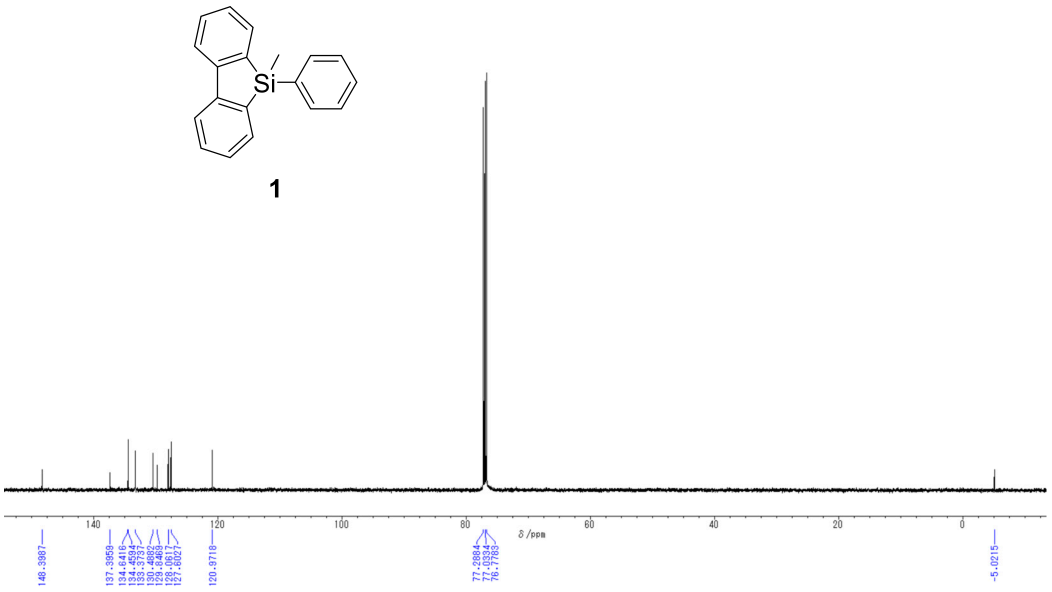

Figure S13. ^{13}C{^{1}H}-NMR of **1**.

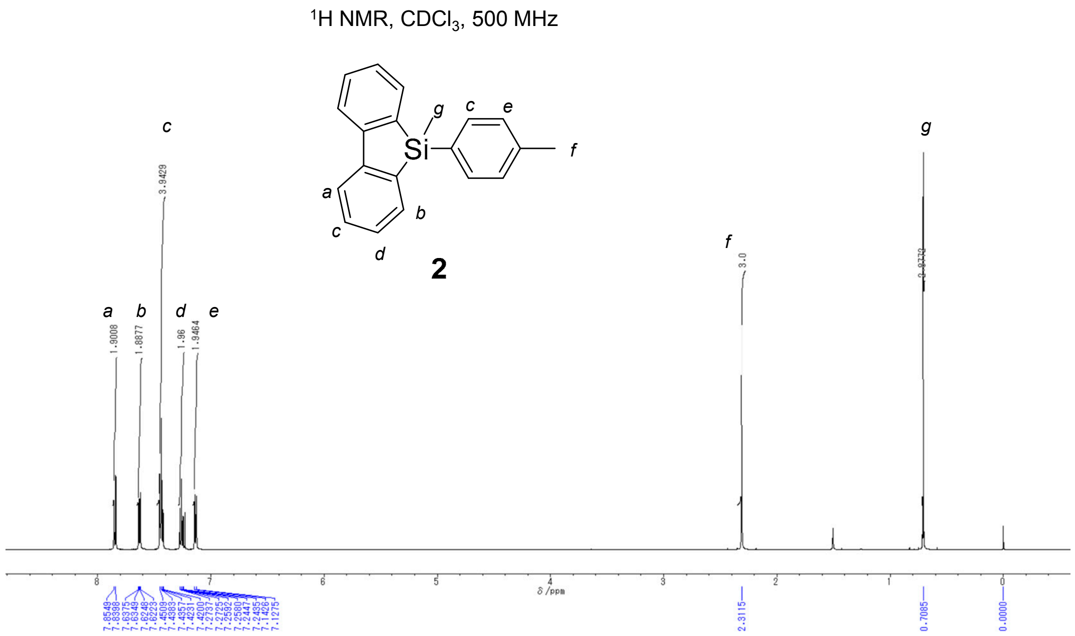

Figure S14. ^{1}H-NMR of **2**.

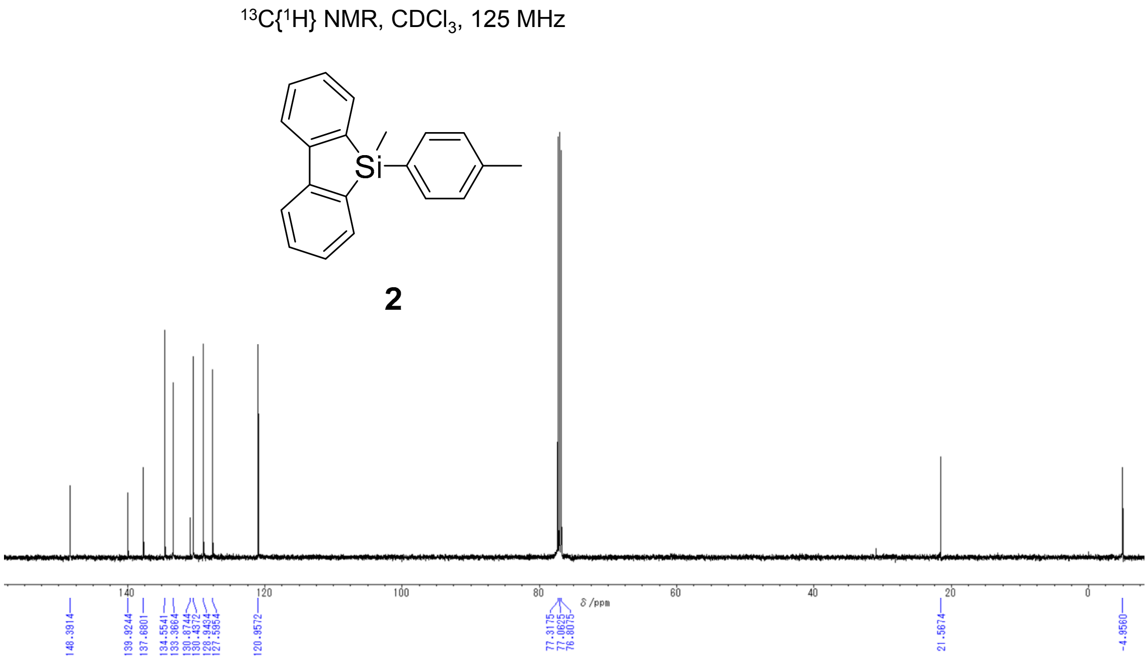

Figure S15. ^{13}C{^{1}H}-NMR of **2**.

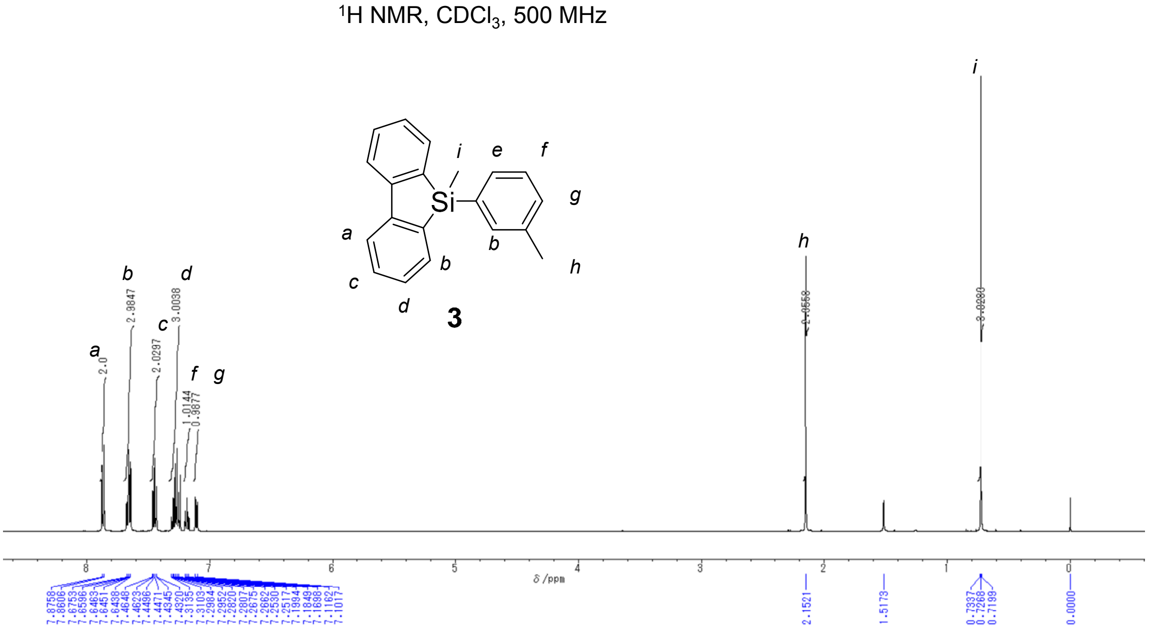

Figure S16. ^{1}H-NMR of **3**.

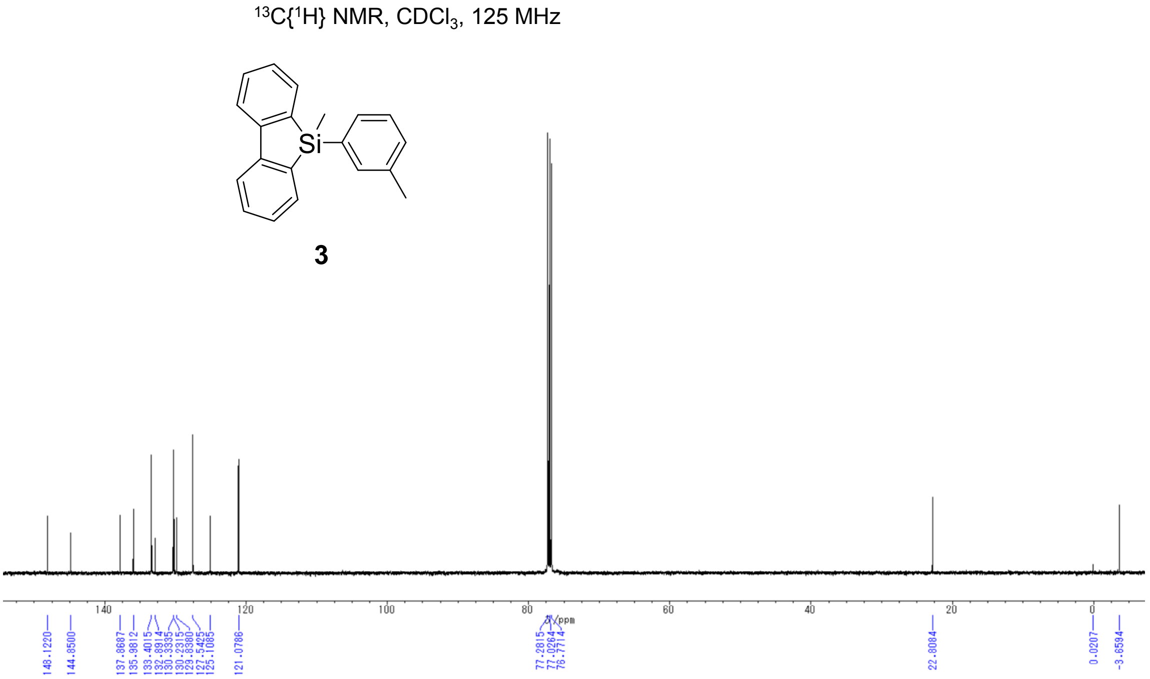

Figure S17. ^{13}C{^{1}H}-NMR of **3**.

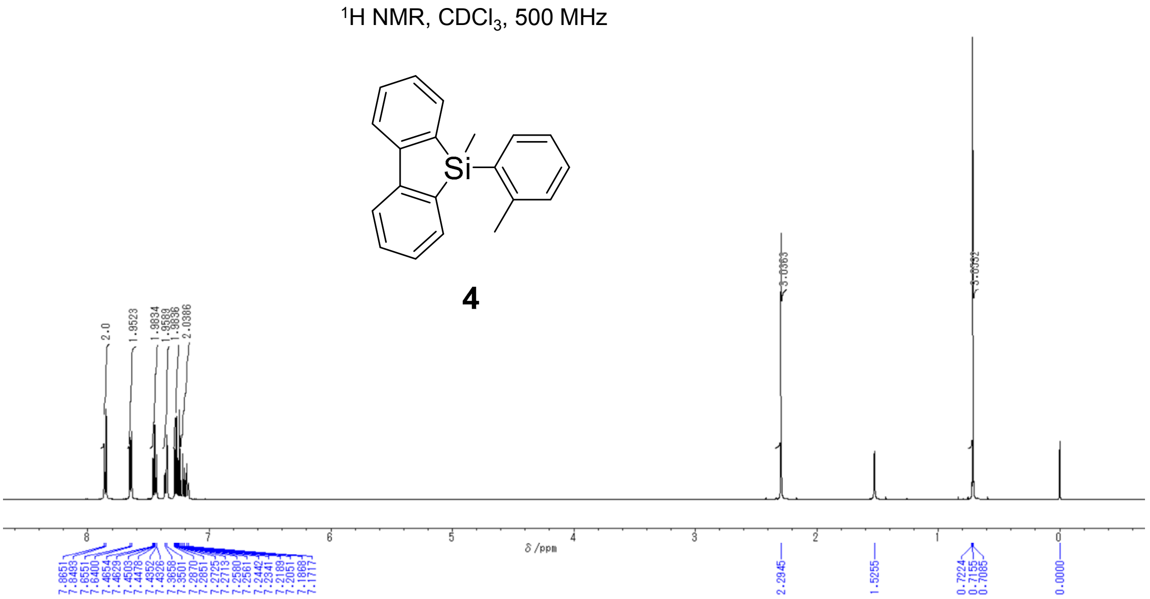

Figure S18. ^{1}H-NMR of **4**.

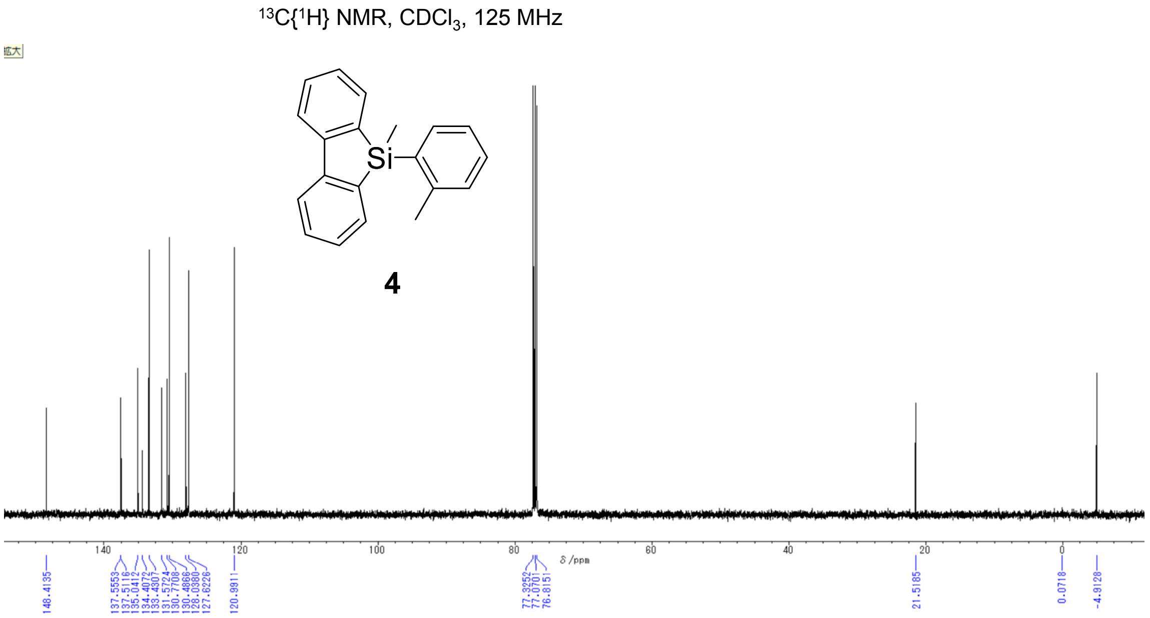

Figure S19. ^{13}C{^{1}H}-NMR of **4**.

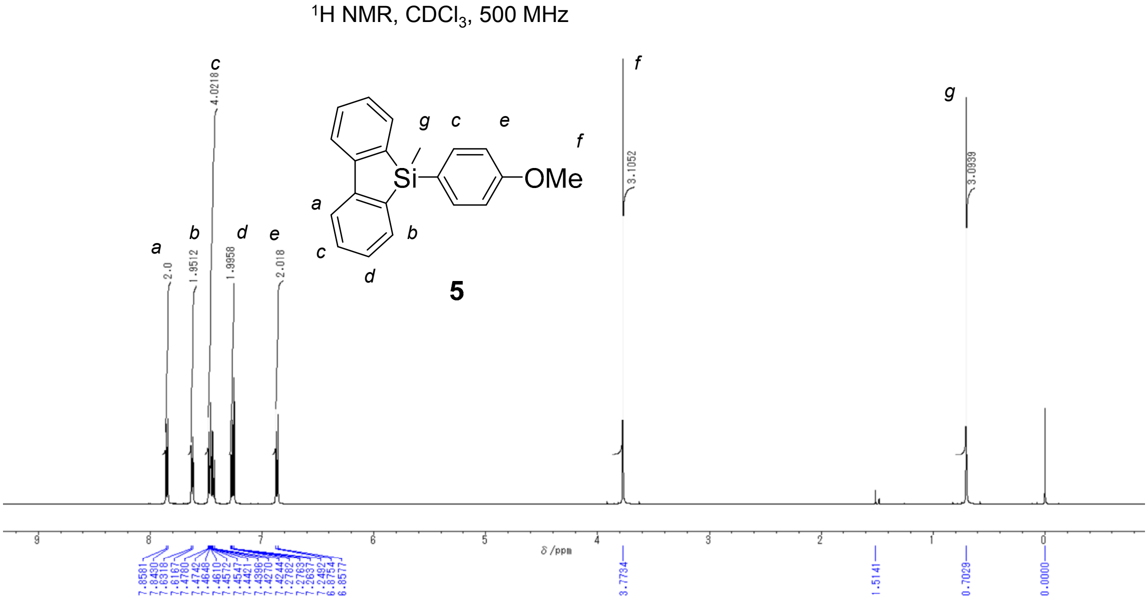

Figure S20. ^{1}H-NMR of **5**.

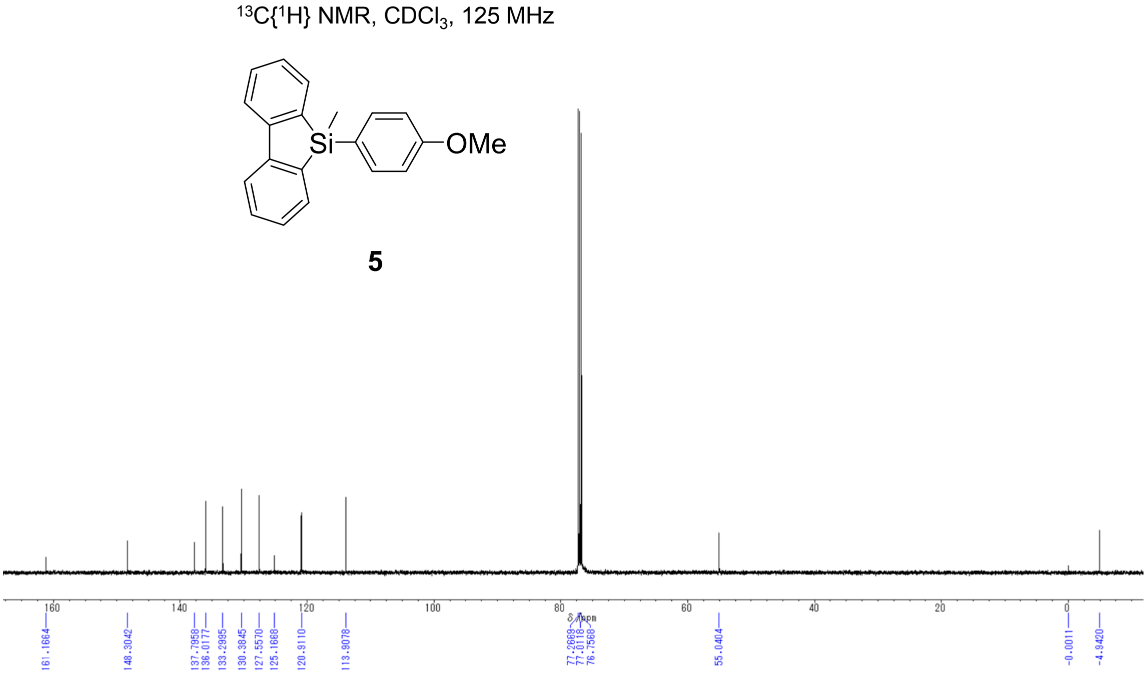

Figure S21. ^{13}C{^{1}H}-NMR of **5**.

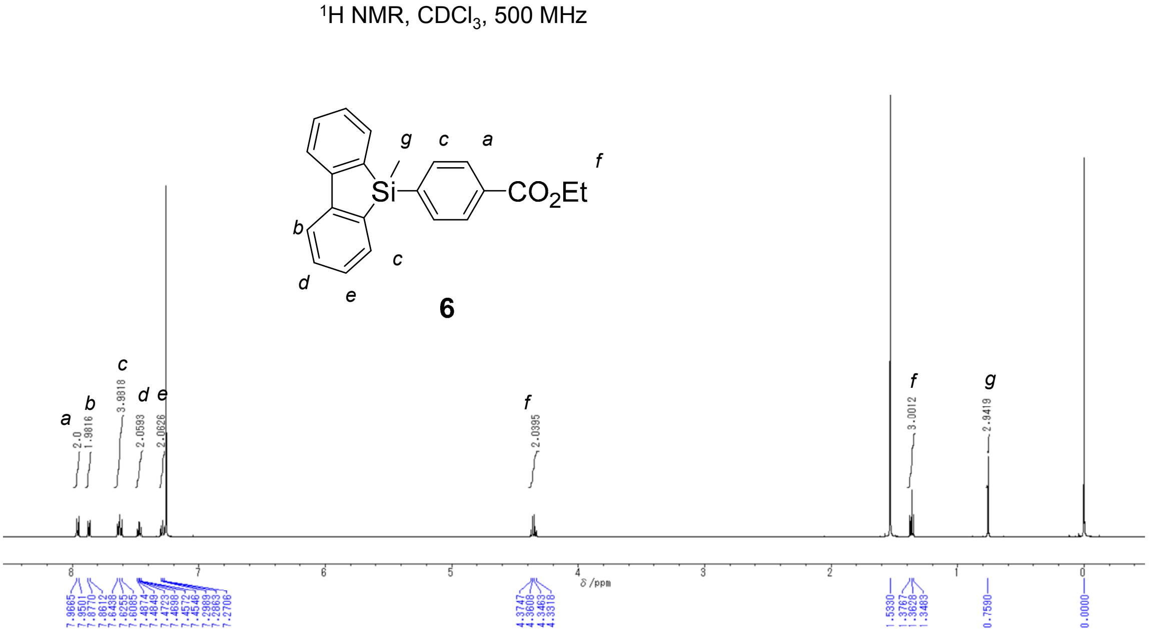

Figure S22. ^{1}H-NMR of **6**.

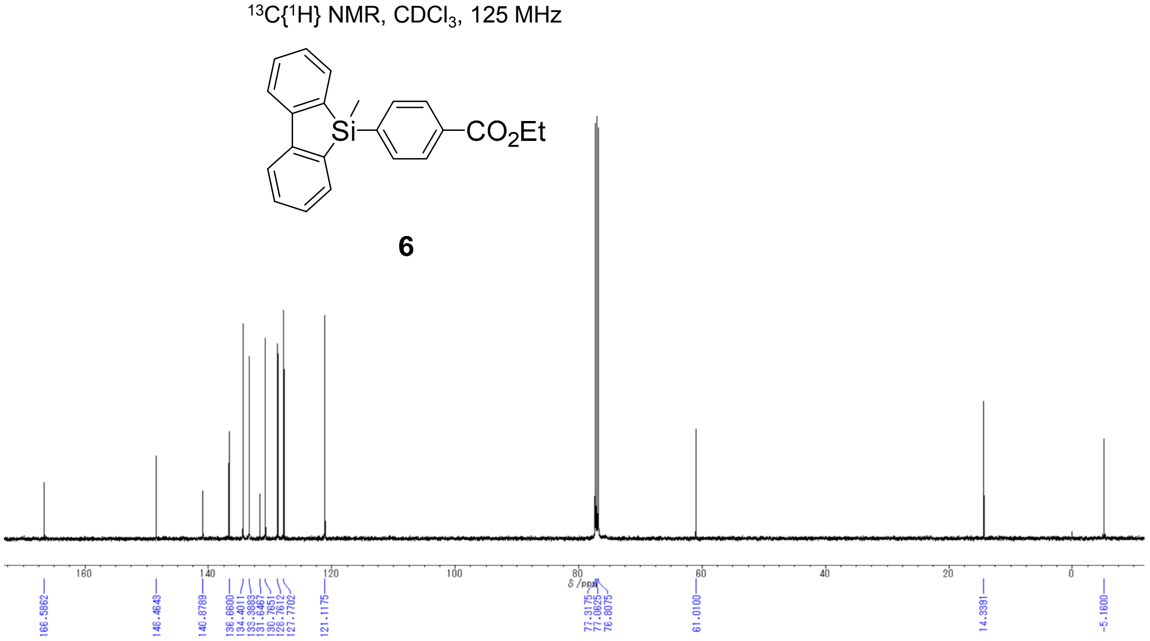

Figure S23. ^{13}C{^{1}H}-NMR of **6.**

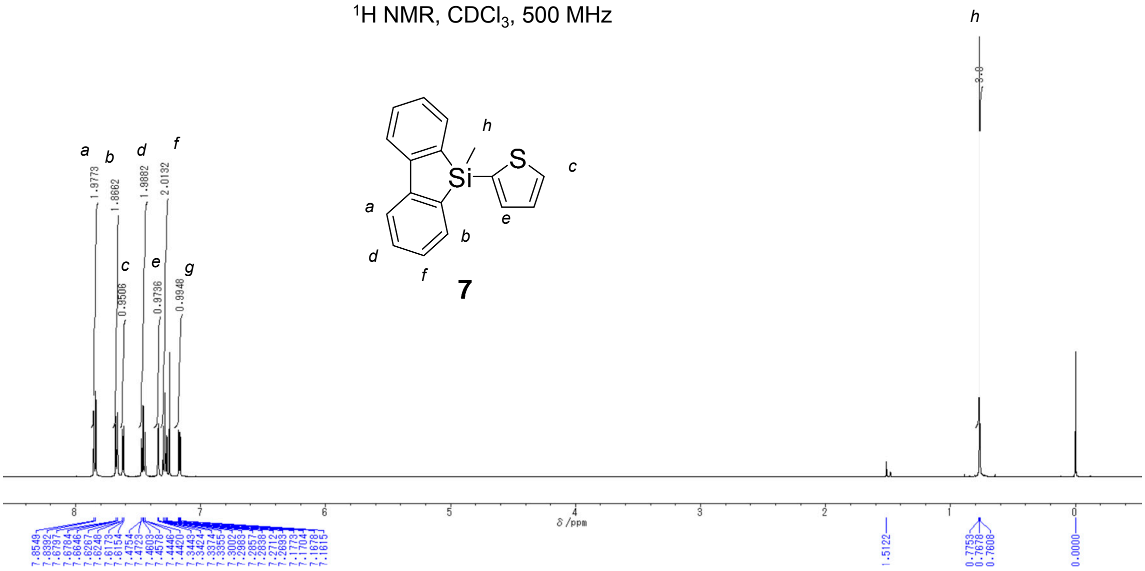

Figure S24. ^{1}H-NMR of **7**.

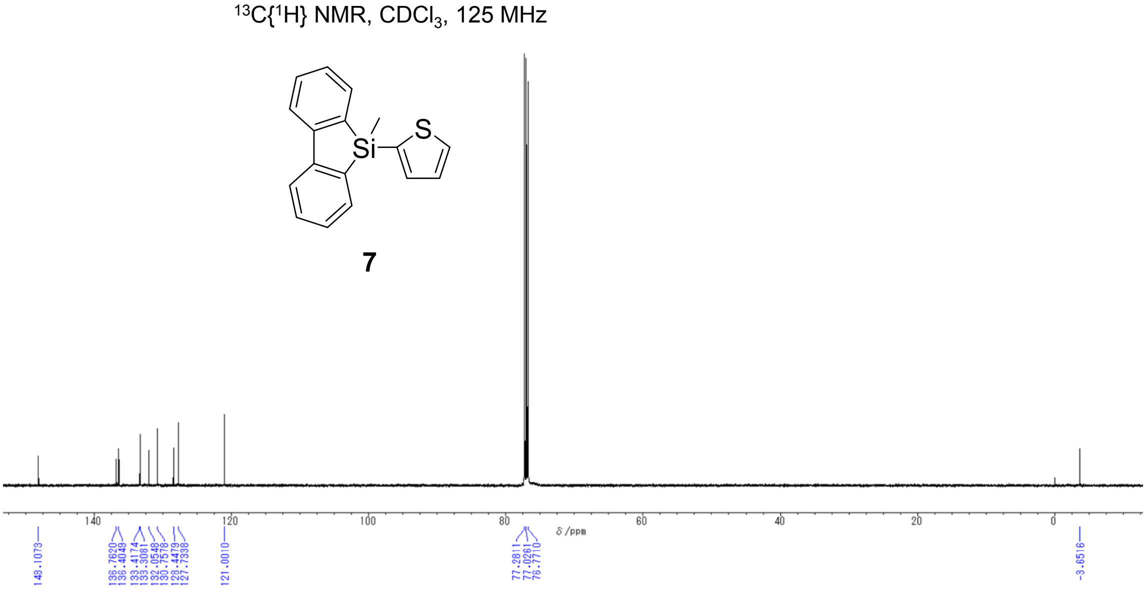

Figure S25. ^{13}C{^{1}H}-NMR of **7**.

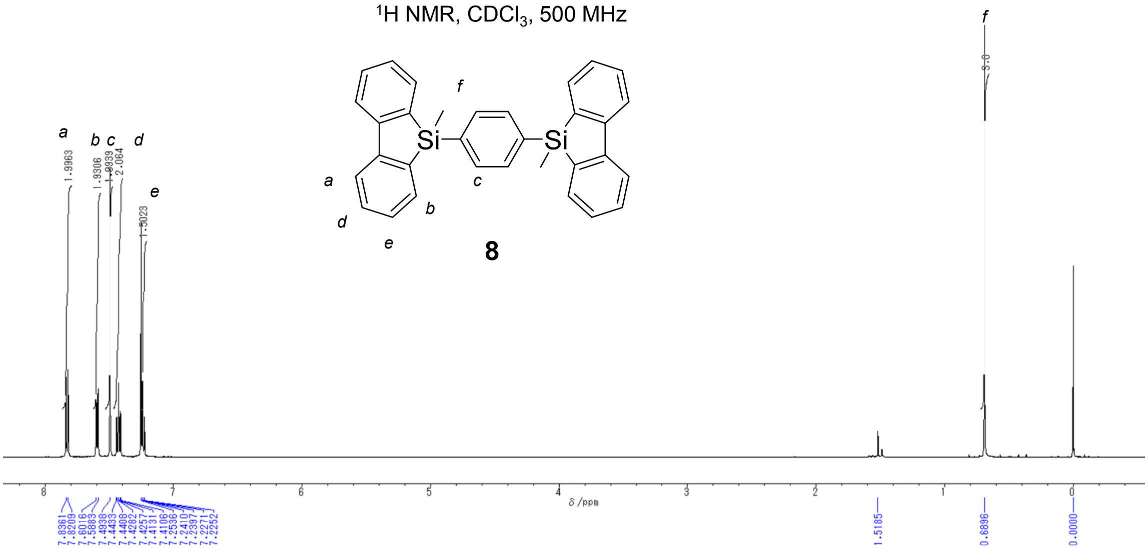

Figure S26. ^{1}H-NMR of **8**.

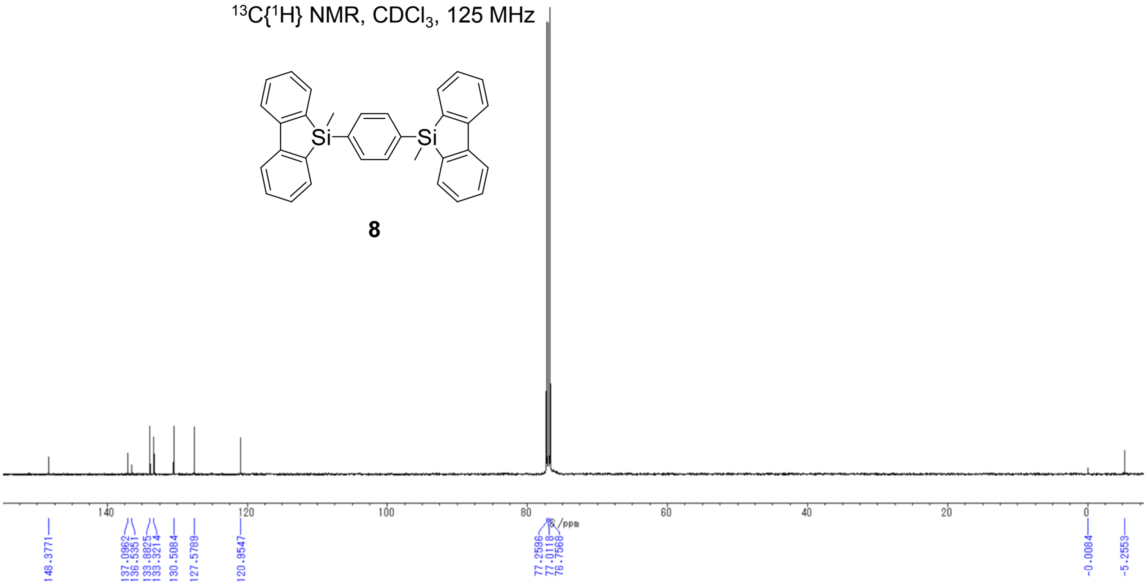

Figure S27. ^{13}C{^{1}H}-NMR of **8**.

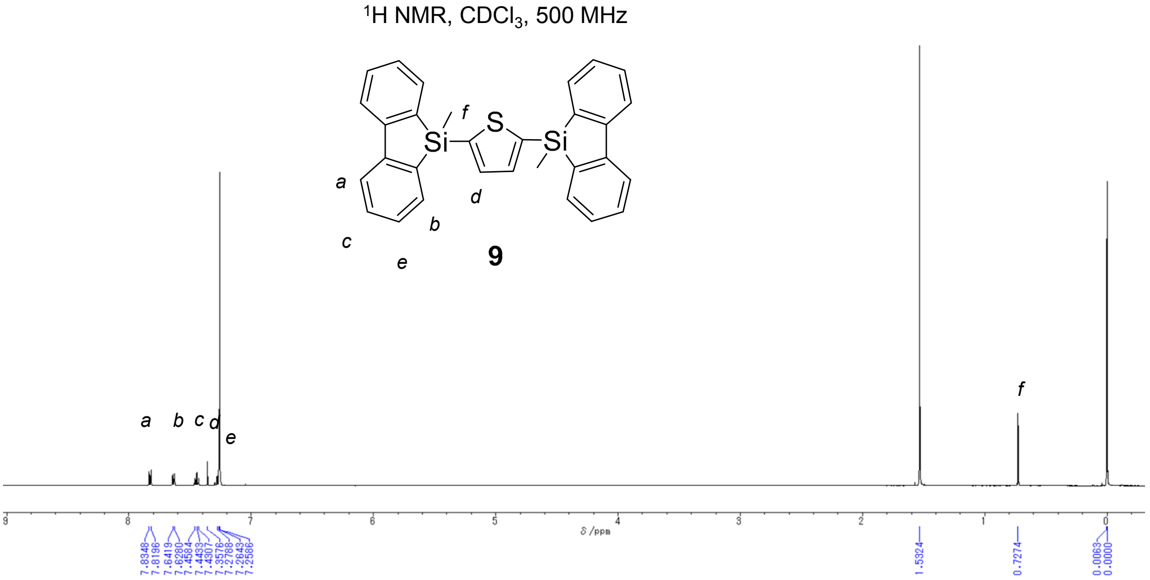

Figure S28. ^{1}H-NMR of **9**.

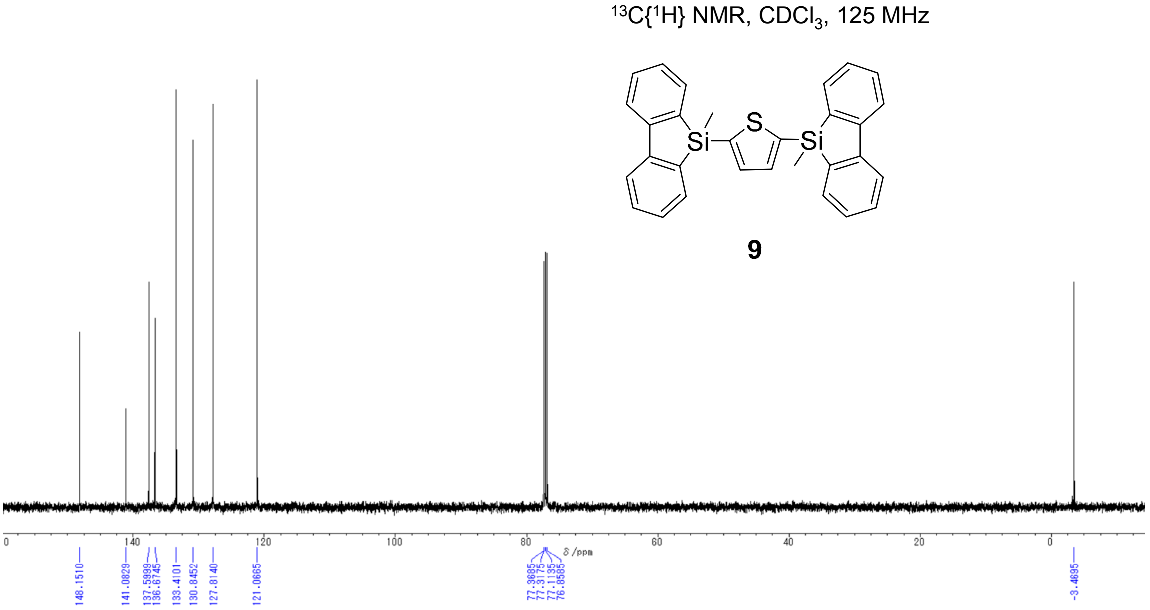

Figure S29. ^{13}C{^{1}H}-NMR of **9**.

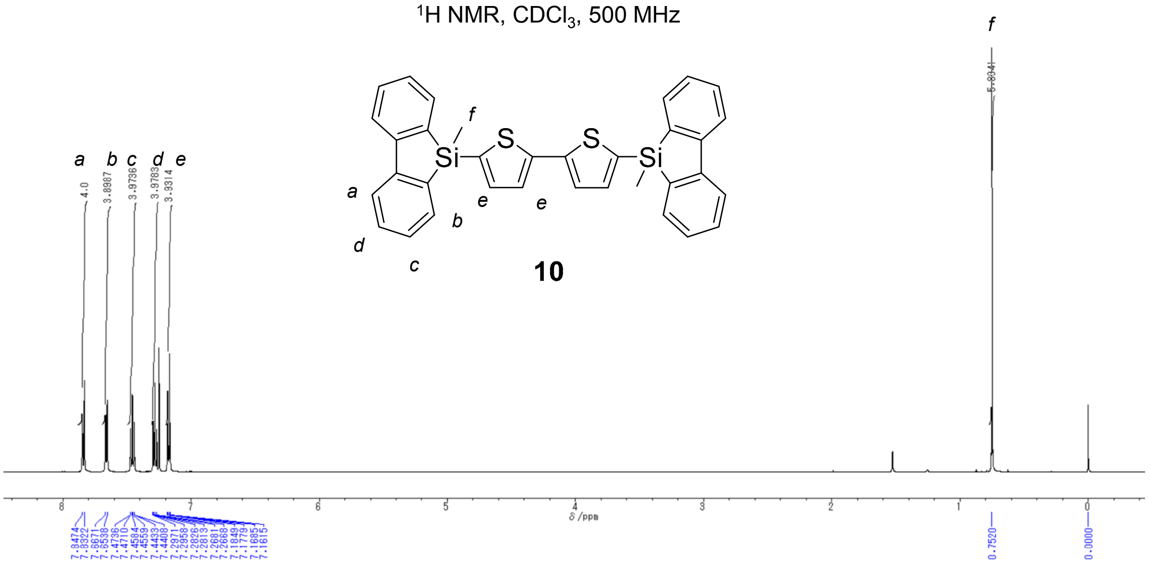

Figure S30. ^{1}H-NMR of **10**.

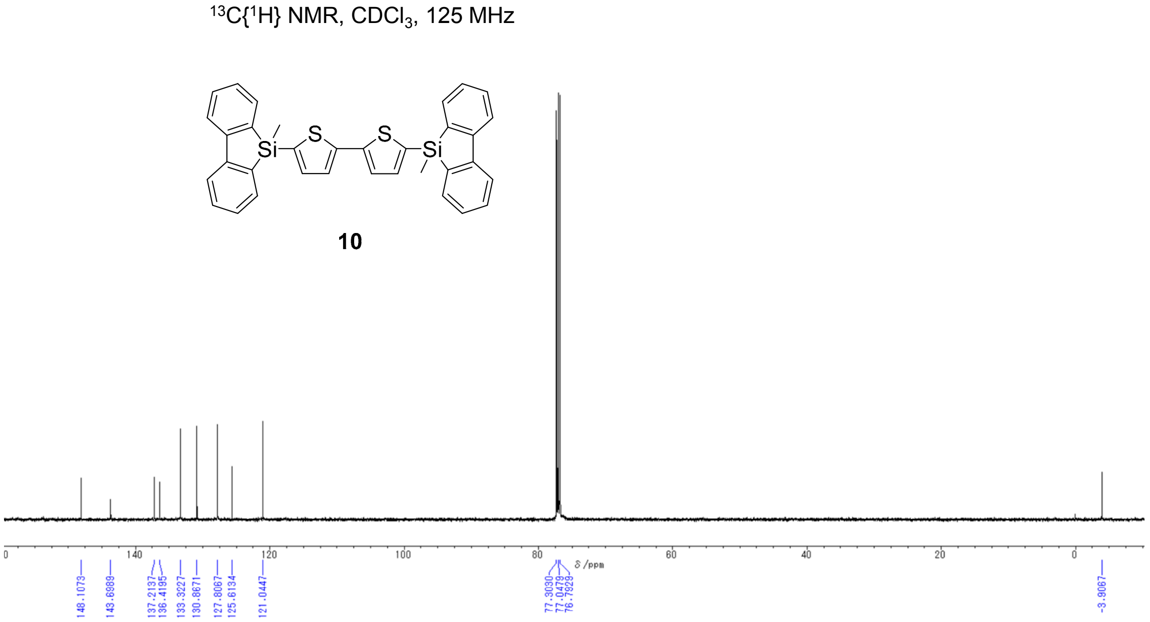

Figure S31. ^{13}C{^{1}H}-NMR of **10**.
